# The Vehicles of Calcium Hydroxide Pastes Interfere with Antimicrobial Effect, Biofilm Polysaccharidic Matrix, and Pastes’ Physicochemical Properties

**DOI:** 10.3390/biomedicines10123123

**Published:** 2022-12-03

**Authors:** Victor Feliz Pedrinha, Maricel Rosario Cardenas Cuellar, Mirela Cesar de Barros, Pedro César Gomes Titato, Mohammad-Ali Shahbazi, Prashant Kumar Sharma, Flaviana Bombarda de Andrade

**Affiliations:** 1 Department of Operative Dentistry, Endodontics and Dental Materials, Bauru School of Dentistry, University of São Paulo, Bauru 17012-901, SP, Brazil; 2 Department of Biomedical Engineering, University Medical Center Groningen, University of Groningen, 9713 GZ Groningen, The Netherlands

**Keywords:** antimicrobial action, biofilm, calcium hydroxide, endodontics, extracellular matrix, intracanal medication, physicochemical properties

## Abstract

The objective of the present study was to investigate the pH, volumetric alteration, antimicrobial action, and effect on biofilm matrix polysaccharides of calcium hydroxide (CH) pastes with different vehicles available in endodontics: CH + propylene glycol (CHP), UltraCal XS^®^, Metapaste^®^, and Metapex^®^. The pH was analyzed at different time intervals using a pH meter. For volumetric alteration, a microtomographic assay was performed before and after immersion in water. *Enterococcus faecalis* was chosen for microbiological tests. The bacterial viability and extracellular matrix were quantified with direct contact evaluation (dentin blocks) and at the intratubular level (dentin cylinders) using LIVE/DEAD BacLight and Calcofluor White dyes via confocal laser scanning microscopy (CLSM). Kruskal–Wallis and Dunn’s tests were used to analyze pH and direct contact assays, while one-way ANOVA and Tukey tests were used to analyze volumetric alteration and intratubular decontamination (α = 0.05). Higher pH values were obtained during the initial days. Volumetric alterations were similar in all groups. Lower bacterial viability was obtained for dentin blocks and cylinders when CH pastes were used. UltraCal XS and Metapex had lower values for the extracellular matrix. The pH of all CH pastes decreased with time and did not promote medium alkalization for up to 30 days. CH paste can reduce bacterial viability through direct contact and at an intratubular level; however, UltraCal XS and Metapex are involved with lower volumes of extracellular matrices.

## 1. Introduction

The use of calcium hydroxide (CH) pastes as intracanal medication between sessions of endodontic treatment is usually the preferred approach to complement disinfection or to avoid the possibility of reinfection [1]. CH provides alkalinization of the internal surfaces of the roots and neutralizes acidic products from the inflammatory process, facilitating repair and antimicrobial activity [2,3]. *Enterococcus faecalis* (*E. faecalis*), a Gram-positive bacterium, can survive in alkaline environments and is associated with persistent endodontic infections [4]; however, when in direct contact with CH at a pH of 12, this species is eliminated [5]. Therefore, to exert antimicrobial effects, CH must be in direct contact with the microbiota present in the root canal system, including all ramifications and dentinal tubules.

Vehicles associated with CH are known to be harmless to microorganisms; however, they can influence the properties of these medications [2,3,6]. The vehicles commonly used are water-soluble (aqueous and viscous) due to their greater ionization and diffusion through tissues 6. UltraCal XS paste (Ultradent Products, Inc., South Jordan, UT) consists of 35% CH in an aqueous matrix of methylcellulose, making it an aqueous vehicle. Metapaste (Meta Biomed Co., Ltd., Chungbuk, Korea) contains polypropylene glycol, a viscous vehicle, and barium sulfate as a radiopaque agent [7]. There are also non-water-soluble or oily vehicles, such as those that use silicone oil, as presented in Metapex (Meta Biomed Co., Ltd., Chungbuk, Korea), which also uses iodoform as a radiopaque agent [8]. However, these CH pastes have not been studied in terms of their properties.

Many studies have simulated direct contact with planktonic bacteria [9,10,11]. However, most bacteria were found in biofilms in vivo. Furthermore, studies are limited because the extracellular polysaccharide matrix, which provides resistance to microorganisms, has not been quantified. In another situation, the surface of the root canals may harbor the remaining microorganisms after chemomechanical procedures due to their small space compared to the lateral canals, branches, and isthmus of the root canal system; therefore, it is important to conduct investigations at the intratubular level [12,13,14,15]. Therefore, an analysis of the intratubular activity of CH pastes is important for further extrapolation to a clinical scenario.

Furthermore, the antimicrobial action of CH pastes is mainly related to their alkaline pH [1,6]. A high volumetric loss can interfere with direct contact between the paste and bacteria, making it impossible for the paste to reach a high pH within the root canal system [1,3]. In this context, studies are necessary to associate the properties of pH, volumetric alteration, antimicrobial action, and extracellular matrix biofilm reduction with the activity of CH pastes. This study aimed to investigate the properties of commercially available CH pastes, UltraCal XS, Metapaste, and Metapex, considering the different vehicles in which these medications are present. The null hypothesis was that there are no significant differences in physicochemical properties, antimicrobial action, or extracellular matrix biofilm reduction between commercial pastes with different vehicles.

## 2. Materials and Methods

This study was registered by the Animal Research and Ethics Committee of the local university (registration number: 016/2019) for the use of extracted bovine teeth.

For each assay performed, four CH pastes were investigated:

1. CH + propylene glycol (CHP), mixed in a ratio of 3:1 (powder weight/vehicle weight).
2. UltraCal XS: CH + methylcellulose + barium sulfate (Ultradent Products Inc., South Jordan, UT, USA).
3. Metapaste: CH + polypropylene glycol + barium sulfate (MetaBiomed Co., Ltd., Chungbuk, Korea).
4. Metapex: CH + silicone oil + iodoform (MetaBiomed Co., Ltd., Chungbuk, Korea).

### 2.1. pH Analysis

Acrylic resin maxillary incisors (*n* = 10 per group) with a simulated artificial root canal and standardized foramen were instrumented with type-K endodontic files (Dentsply/Maillefer; Ballaigues, Switzerland) with a diameter of 0.40 mm. Four different CH pastes were placed in the canal and the coronary access was sealed with Bioplic (Biodynamics, Londrina, Brazil). The apical foramen was the only contact between the medication and the external environment. Each specimen was individually immersed in containers containing 10 mL of distilled water and, after intervals of 7, 15, and 30 days, transferred to new plastic containers with the same amount of distilled water. During this period, the specimens were incubated at 37 °C. The pH of the solutions was analyzed using a pH meter (Model 371; Micronal, São Paulo, SP, Brazil). The measurements were performed in the solutions in which the specimens were stored. This method is based on previous studies [1,3,16].

### 2.2. Micro-Computed Tomographic Volumetric Analysis

For volumetric analysis, 40 acrylic resin upper incisors (*n* = 10) with artificial root canals and foramen were instrumented with endodontic files (Dentsply/Maillefer, Ballaigues, Switzerland) up to a diameter of 0.40 mm (K-File #40). The specimens were then filled with different CH pastes until the root canals were filled without air bubbles, as verified by microtomography (SkyScan 1174v2; SkyScan, Kontich, Belgium). The open-access crown of each specimen was then sealed with Bioplic (Biodynamics, Londrina, Brazil), and CT scans were initiated (SkyScan 1174v2; SkyScan, Kontich, Belgium).

The image capture parameters were as follows: voxel size, 19.70 µm; rotation step, 0.5°; and rotation, 360°. Each scan consisted of 373 TIFF images of 1024 × 1304 pixels. After scanning, the samples were completely immersed in plastic containers containing 10 mL of distilled water and stored at 37 °C. After 15 days, the specimens were removed from their containers, and without any washing process, a new scan was performed with the same parameters mentioned above. Digitized images were reconstructed, and the volumes (mm^3^) of CH pastes were measured using CTan software (CTan v1.11.10.0, Sky-Scan). The reduction in values was obtained by subtracting the final value from the initial value. The percentage of volumetric change was calculated by dividing the volume lost by the total value [1,3].

### 2.3. Antibiofilm Direct Contact Test

Recently extracted bovine incisor teeth, acquired by donation from a slaughterhouse, were stored in a 0.1% thymol solution at 4 °C. Fifty dentin blocks were obtained using a 5.0 mm trephine bur (Härte Surgical Instruments, Ribeirão Preto, SP, Brazil) adapted to a handpiece (Kavo Kerr, Joinville, SC, Brazil). The dentin surfaces were sequentially prepared to obtain standardized specimens according to a previous study [17]. The blocks were left in an ultrasonic tub with 17% ethylenediaminetetraacetic acid (EDTA) and 5% sodium thiosulfate (Fórmula e Ação, São Paulo, SP, Brazil) for 5 min each to remove the smear layer. The blocks were washed in distilled water and sterilized at 121 °C for 20 min.

All microbiological procedures were performed in a laminar flow chamber under aseptic conditions (VecoFlow Ltd., Campinas, SP, Brazil). For the biofilm of *E. faecalis*, the strain stored by freezing ATCC 29212 (American Type Culture Collection) was reactivated in 3 mL of sterilized brain–heart infusion (BHI) (Oxoid, Basingstoke, UK). Purity was confirmed by colony morphology and Gram staining (Oxoid, Basingstoke, UK). The culture was adjusted according to McFarland standard #1 (3 × 10^8^ CFU/mL) using an SF325NM spectrophotometer (Bel Photonics do Brasil Ltd.a., Osasco, SP, Brazil) and incubated at 37 °C for 7 h to reach exponential bacterial growth.

The dentin surfaces were infected using 24-well plates, in which *E. faecalis* inoculum (100 µL), 1 dentin block, and 900 µL BHI were inserted individually into each well of the plate. Plates were incubated aerobically at 37 °C for 7 days, and the BHI was renewed every day. After the incubation period, the infected samples were washed with 1 mL of distilled water to remove loosely adhered bacteria. The blocks were then randomly assigned to four groups (*n* = 10) according to the experimental pastes: CHP, UltraCal XS, Metapaste, and Metapex. In addition, eight specimens were used as positive controls (no treatment) to determine bacterial viability before administering medications, and two specimens were used as negative controls. The dentin blocks with biofilms were immersed in experimental medications and incubated at 37 °C for 7 days for the direct contact test.

After the incubation period, the medications were removed using 10% citric acid (Specífica Pharmacy, Bauru, SP, Brazil) and distilled water to inactivate the residual alkaline pH and remove loosely adherent planktonic bacteria, respectively. The specimens with biofilms were then stained with 10 μL of the LIVE/DEAD^®^ BacLight viability kit (Molecular Probes, Eugene, OR, USA) for 10 min to determine biovolume and microbial viability and 10 μL of Calcofluor White M2R dye (Merck, Darmstadt, Germany) for 1 min to identify the extracellular matrix. The LIVE/DEAD^®^ kit contains SYTO 9 dye, which stains live cells with a green pigment, and propidium iodine dye, which stains dead cells with a red pigment. Calcofluor White M2R is a fluorescent blue dye that is used to stain extracellular polymeric matrix (ECM) substances.

The specimens were washed again, placed on a glass slide with immersion oil, and observed under a Leica TCS-SPE confocal microscope (Leica Microsystems GmbH, Mannheim, Germany) at 40× magnification. The images were converted to the “tiff” format using Leica Application Suite-Advanced Fluorescence software (LAS AF, Leica, Mannheim, Baden-Württemberg, Germany), exported, and analyzed using Leica LAS X Life Science software (Leica Microsystems GmbH, Mannheim, Germany). Four sequential images were obtained from different parts of each bovine dentin block. The images were obtained using 23 deep sections of 1 μm step size in 1024 × 1024 pixels. The formula was applied according to previous studies to obtain the percentage of bacterial viability [15,18]. The volume of the extracellular matrix biofilm was obtained in µm^3^.

### 2.4. Antibiofilm Intratubular Test

Bovine incisors were decoronated, and the 5 mm apical end of the roots was removed using an Isomet saw (Isomet^®^ 1000, Buehler Ltd., Lake Bluff, IL, USA) with a diamond disc at 250 rpm under continuous irrigation. Standardized 8 mm length dentin cylinders were obtained. The inside diameters of the dentin cylinders were prepared using a Gates Glidden drill size 4 (Dentsply Maillefer, Ballaigues, Switzerland) to standardized diameter of 1.1 mm according to previous reports [19,20,21]. The dentin cylinders were subjected to sequential ultrasonic baths with 1% sodium hypochlorite (NaOCl) (Fórmula e Ação, São Paulo, SP, Brazil), 17% EDTA, 5% sodium thiosulfate (Fórmula e Ação, São Paulo, SP, Brazil), and distilled water for 10 min each. Finally, the specimens were covered with red nail polish (Colorama, Rio de Janeiro, RJ, Brazil) and sterilized in an autoclave (Cristófoli, Campo Mourão, PR, Brazil) at 121 °C for 24 min.

The specimens were placed in microtubes (Eppendorf, Hamburg, Germany) with sterile BHI culture medium and subjected to an ultrasonic bath for 10 min to maximize the penetration of the culture broth into the dentinal tubules [1,13,15,19,20,22]. A culture of *E. faecalis* (ATCC 29212) was used for contamination, and the bacterial suspension was adjusted to McFarland standard #1 (3 × 10^8^ CFU/mL). Each dentin cylinder was placed in a 1.5 mL microtube, which was filled with 1 mL of the *E. faecalis* suspension and incubated at 37 °C for 5 days, following the Ma et al. sequence of centrifugation steps [22] and Andrade et al. protocol [23], which has previously been reproduced [1,13,15,19,20,24].

The specimens were randomly assigned according to the intracanal medication used (*n* = 10): CHP, UltraCal XS, Metapaste, and Metapex. In addition, eight specimens were randomly selected as positive controls to confirm intratubular contamination, and two additional specimens were selected as negative controls to confirm sterility. The specimens were placed in a sterilized metal device inside the laminar flow chamber, and CH pastes were inserted with the help of a K-file #40 (Dentsply Maillefer, Ballaigues, Switzerland) until complete filling. After insertion of the medication, a temporary sealant (Coltosol^®^, Coltene, Rio de Janeiro, RJ, Brazil) was applied to the ends of the dentin cylinders. The medication remained in the specimens, which were kept in sterilized microtubes with small pieces of gauze soaked in sterilized distilled water at 37 °C for 15 days.

After this period, the temporary sealant was removed and the CH pastes were removed in the same manner as described for the dentin blocks. The specimens were sectioned longitudinally using an Isomet machine with a diamond disk under constant irrigation with a sterile saline solution. The smear layer resulting from the cut was removed by immersion in 17% EDTA for 3 min, washed with distilled water, and dried using sterilized absorbent paper [1,13,15,19,20,24]. The dentin cylinders were stained with 30 μL of the LIVE/DEAD^®^ BacLight viability kit for 20 min and 30 μL of Calcofluor White M2R dye for 2 min. Confocal laser scanning microscopy (CLSM) analysis was performed following the same specifications as direct contact test for image acquisition; however, for intratubular analysis, eight sequential images were obtained from each sample, four near the main root canal (superficial area) and four further away from the main canal (deep area) [13,19,20]. Then, the percentages of bacterial viability and extracellular matrix biofilms were quantified.

### 2.5. Statistical Analysis

Data distribution was assessed using the Shapiro–Wilk normality test. After normality evaluation, volumetric alteration and intratubular data showed a parametric distribution, while pH and direct contact data showed a non-parametric distribution. Therefore, one-way ANOVA followed by Tukey’s test was performed for volumetric alteration and intratubular analysis. Kruskal–Wallis test, followed by Dunn’s test, were performed for pH and direct contact tests. GraphPad Prism 8.0 software (GraphPad, San Diego, CA, USA) was used for the analysis and a significance level of 5% was adopted.

## 3. Results

Table 1 presents the median, minimum, and maximum pH values of the pastes at the analyzed time intervals. At 7 days, the CH pastes showed higher pH values, with a continuous decrease observed at 15 and 30 days.

Table 2 shows the mean and standard deviation for the initial and final volumes of CH pastes and the percentage of volumetric alteration within 15 days. There were no statistically significant differences between the pastes (*p* > 0.05).

**Figure 1:**
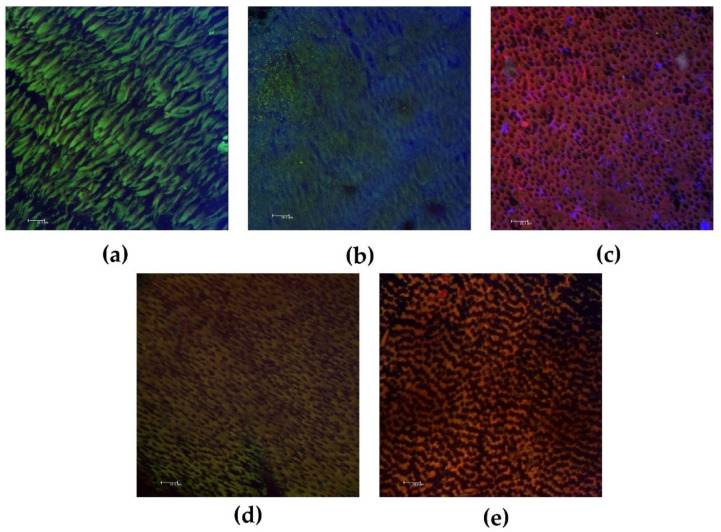
Representative confocal laser scanning microscope images of biofilms after direct contact with calcium hydroxide pastes. (**a**) Control group; (**b**) calcium hydroxide + propylene glycol; (**c**) UltraCal XS; (**d**) Metapaste; (**e**) Metapex. Viable bacteria are indicated in green, and non-viable bacteria are indicated in red. The extracellular matrix biofilms are indicated in blue. Magnification: 40×. Bars: 20.0 μm.

Table 3 presents the median, minimum, and maximum values of the percentage of bacterial viability and extracellular matrix biofilm volume. UltraCal XS, Metapaste, and Metapex treatments showed lower bacterial viability (*p* < 0.05). Furthermore, UltraCal XS and Metapex showed a lower volume of biofilm extracellular matrix (*p* < 0.05). Representative images of biofilms after direct contact with CH pastes are shown in Figure 1.

**Figure 2:**
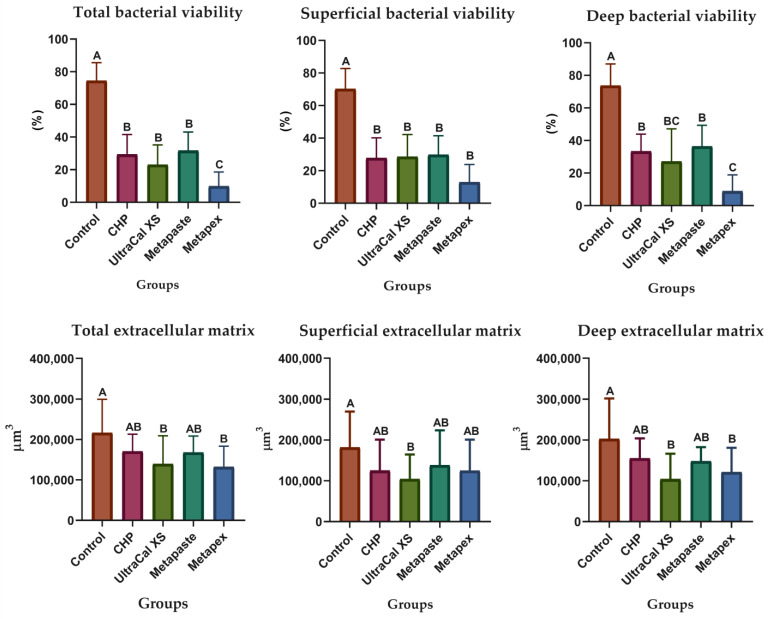
The percentage (%) of viable bacteria and the volume (µm^3^) of extracellular matrix biofilm of each group in the total, superficial, and deep areas of the specimens. Different capital letters indicate a statistically significant difference between medications in the same area. The bars indicate the mean, and the vertical dashes above the bars indicate the standard deviation. CHP, calcium hydroxide + propylene glycol.

Figure 2 presents the graphs of the percentage of bacterial viability and biofilm extracellular matrix volume after intratubular evaluation using CLSM. The Metapex showed lower bacterial viability percentages (*p* < 0.05) when evaluating the total area of the specimens. The investigated CH pastes showed lower values of bacterial viability in the superficial region but did not differ from each other; however, in the deep area, the bacterial viability promoted by the UltraCal XS and Metapex pastes showed better results, mainly Metapex (*p* < 0.05).

**Figure 3:**
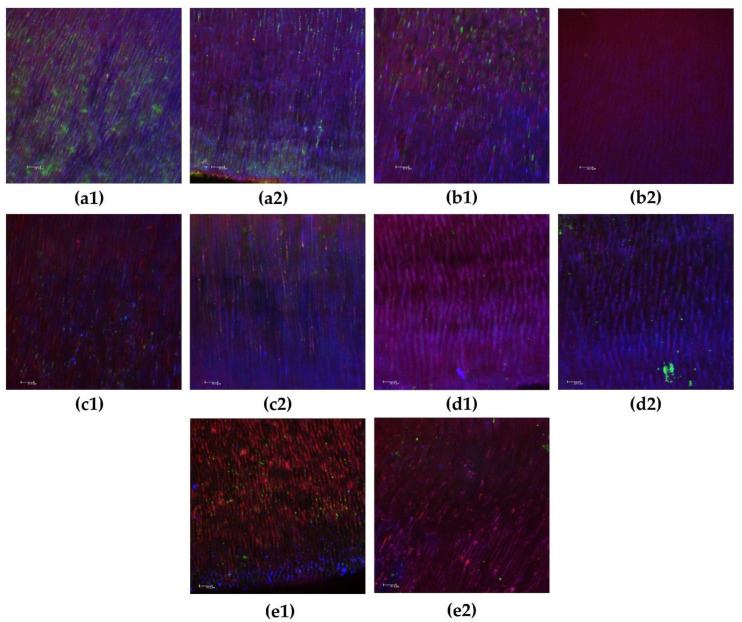
Representative confocal laser scanning microscope images of intratubular biofilms after 15 days with calcium hydroxide pastes. (**a1**,**a2**) Control group in the superficial and deep areas, respectively; (**b1**,**b2**) calcium hydroxide + propylene glycol in the superficial and deep areas, respectively; (**c1**,**c2**) UltraCal XS in superficial and deep areas, respectively; (**d1**,**d2**) Metapaste in superficial and deep areas, respectively; (**e1**,**e2**) Metapex in superficial and deep areas, respectively. Viable bacteria are indicated in green, and non-viable bacteria are indicated in red. Extracellular matrix biofilms are indicated in blue. Magnification: 40×. Bars: 20.0 μm.

In relation to biofilm extracellular matrix volume, although the CH pastes showed lower values, only UltraCal XS and Metapex differed from the control group in total and deep areas (*p* < 0.05). Representative images of biofilms in superficial and deep intratubular dentin after CH paste treatment are shown in Figure 3.

## 4. Discussion

Commercially, CH pastes are available with different vehicles in their composition, as CH powder needs to maintain an alkaline medium through ionic release [16]. Therefore, it is essential to evaluate the physicochemical characteristics, antimicrobial action, and effect of these medications on the extracellular matrix biofilm to better understand the influence of vehicles on CH properties. Given the present results, the null hypothesis of this study was rejected due to the significant differences observed between the CH pastes in the assays.

The pH of the CH pastes depended on the ratio of CH to vehicle. A higher mixing ratio of CH powder with propylene glycol or other vehicles (>3) could result in pH > 11. However, a large amount of CH powder mixed with a small amount of vehicle could result in thick intracanal medication and compromise important aspects of the CH paste, such as fluidity and penetrability within the root canal system. High pH values of CH promote antibacterial activity through an irreversible enzymatic reaction [25]. Previous studies reported that aqueous and viscous CH pastes maintained an alkaline pH for up to 4 weeks in cylindrical polyethylene tubes filled with CH pastes [5]. However, in a clinical situation, the alkalinization and antimicrobial action of pastes can be impaired by associating this with an intense buffer system in the periapical region. In this study, the pH results for 7 days showed more alkaline values with a continuous decrease, while within 15 days only the Metapaste group showed alkalinity. Within 30 days, all pastes showed values below the recommended value, corroborating other studies [3,26]. Furthermore, the use of standardized acrylic teeth aims to simulate clinical conditions and favor the pH analysis in the apical and periapical regions after different times of contact with drugs [1,3]. In cases of teeth with pulp necrosis and periapical lesions, foraminal cleaning is necessary, and the literature recommends enlargement to at least caliber #40, even for a low incidence of post-operative pain [27].

The literature recommends that a pH of approximately 8.6−10.3 is necessary for the biological action of CH pastes, which favors the activation of alkaline phosphatase and helps the mineralization process [28]. In contrast, a pH above 11 can result in cytotoxicity in the periapical region [29]. Histopathological analysis showed evidence of better apical and periapical tissue repair with the formation of a mineralized apical barrier in the specimens treated with CH pastes, especially in periods of 15 and 30 days, and poor results when it was left in place for only 7 days [30,31]. An accentuated decrease in inflammation at 30 days was previously reported and was compared to the initial days after CH medication was filled in the root canals [30,31]. Therefore, an initial alkaline pH is important to induce periapical tissue repair through more favorable mineralized apical barrier formation. In this study, we observed a high initial pH that decreased with time.

Propylene glycol, as a viscous vehicle, induces a more favorable release of calcium and hydroxyl ions than aqueous vehicles, such as distilled water and saline solution, with a higher pH [5,16,26]. In this study, the CHP paste was not statistically different from UltraCal XS, considering the different assays performed, which had not yet been reported in the literature. UltraCal XS contains an aqueous matrix of methylcellulose as the vehicle. Regardless of vehicle type, methylcellulose is a great option for antibiotic delivery in many studies, considering its biocompatible nature [32,33,34]. This probably contributed to the decrease in bacterial viability and the reduction of the extracellular biofilm matrix in direct contact and intratubular assays.

Pastes with more fluid consistency, such as water-soluble vehicles (aqueous or viscous), can lead to high pH and calcium release in the periapical tissue [1,3,5,16]. However, over the years, the literature has stated that oily vehicles are insoluble in water, resulting in lower solubility and diffusion of the paste in the tissues. Therefore, CH pastes containing oily vehicles are likely to remain within the root canal longer than pastes containing aqueous or viscous vehicles [29]. However, these studies did not consider that the properties of CH pastes can be determined not only by the vehicle that each one contains, but also by the effect of all of its components, including radiopaque materials and additives. The CH pastes in oily vehicles have not yet been investigated using the methods described in this study. Iodoform is a drug with antibacterial activity [35] and is present in Metapex as a radiopaque agent. A previous study reported that iodoform in contact with *E. faecalis* could destroy the bacterial wall after 7 days, with a maximum peak at 14 days [36]. Furthermore, dentin inhibits the action of iodine compounds [37]. When alkaline CH and acidic iodoform were combined, an alkaline pH was initially observed, but these values rapidly decreased regardless of the vehicle used, eventually establishing an acidic pH [36]. This study corroborates these findings, showing that Metapex had an alkaline pH at 7 days, with a continuous decrease, reaching an acidic pH at 30 days.

A solubility test was performed using computed microtomography to verify the volumetric loss of the pastes after 15 days. This period can be considered satisfactory for the action of CH pastes, as in clinical conditions, it is a great challenge for the patient to remain with a sealed tooth, susceptible to chewing loads and possible fractures for a period longer than 15 days. In a previous study, the CH paste with propylene glycol as the vehicle had the highest percentage of volumetric reduction values [1]. Several studies claim that aqueous, viscous, and oily vehicles have different solubility levels, with aqueous pastes showing higher values and pastes in oily vehicles showing the lowest values according to ionic release analyses [5,28]. However, with the introduction of studies using computed microtomography, volumetric alteration analyses at different time intervals have shown a greater volumetric loss in pastes with viscous vehicles [1,3].

Pastes manipulated with aqueous vehicles reach a dry aspect inside the root canals, which can decrease the ionic release and solubility. Therefore, the hydration of pastes can have a greater influence on solubility than vehicle type [3]. Following this reasoning, just as the viscous vehicle influences the hydration of the pastes, the oily vehicle can also contain its drying, which explains the statistically similar volumetric loss among the investigated CH pastes, considering the 15 days between endodontic treatment sessions. In addition, the volumetric loss of CH pastes can interfere with direct contact of medications with bacteria, making it impossible to reach high pH, impairing the antimicrobial action of the pastes, and facilitating the reinfection of unfilled spaces [1,3]. This may also confirm that a period longer than 15 days may not be very interesting for maintaining the properties of pastes inside the root canals. A viable alternative would be the renewal of medications, depending on the specificity of each clinical situation.

*E. faecalis* was the bacterial species chosen due to its characteristics of being found in secondary and persistent endodontic infections compared to primary infections, its ability to penetrate dentinal tubules, adherence to dentin collagen, and resistance to intracanal disinfection procedures commonly performed, in addition to its organization in biofilms [4,15,38]. Moreover, these bacteria can resist high pH conditions [39].

Direct contact tests evaluate the effect of proximity between tested materials and microorganisms on their viability organized in biofilms, regardless of the solubility and diffusion of the antimicrobial components presented by the tested materials [40]. None of the pastes in the present study completely eliminated bacterial viability. The time required for CH pastes to act optimally, promote the disinfection of the root canal system, and achieve a rapid and significant increase in pH is still unknown [1,3].

Previous studies evaluated the release of ions directly in CH paste or in distilled water with immersed pastes and demonstrated high pH values of approximately 12.5 [3,39], simulating the action of CH pastes in direct contact with biofilms. Most bacteria cannot survive in an extremely alkaline environment, such as that provided by CH, even *E. faecalis* [5]; however, in the form of biofilms, CH pastes show limited antimicrobial capacity [1,3]. In this study, even in direct contact, CHP did not show significant differences from the control group (no treatment), despite lower bacterial viability values, in agreement with previous findings that evaluated CH pastes with viscous vehicles [3]. In this assay, Metapaste (viscous vehicle) showed an extracellular matrix biofilm volume similar to that of the control. Therefore, even at high pH, 7 days may not be a sufficient time for viscous pastes to achieve maximum elimination of bacterial cells in biofilms in a direct contact manner. However, in an intratubular evaluation and 15 days of medication inside the root canal, all CH pastes reduced total bacterial viability.

The application of the intratubular method can enable the investigation of the action of CH pastes on bacteria located deeper in the dentinal tubules, that is, an antimicrobial action at a distance that is different from the direct contact method. These bacteria are probably not involved with large amounts of extracellular matrix, since dentin itself may be its main protection and source of nutrition. If this theory is correct, antimicrobials need to reach/kill them deeply without the need to win/disorganize the matrix.

In this study, Metapex showed the lowest values for bacterial viability in the two assays performed: direct contact and intratubular decontamination. The dentinal tubule disinfectant action of Metapex against *E. faecalis* was observed through colony forming units (CFU)/mL and showed the most effective results [8]. The pH of the Metapex is below that which is effective for killing *E. faecalis*, especially after 7 days, as observed in the present study. However, the superior antimicrobial effects of Metapex may be due to its combination with iodoform and an oily vehicle, which may prolong the action of the medicament [8]. Therefore, we can hypothesize that a synergistic effect occurs between iodoform and CH in relation to antimicrobial activity. Another study showed that oily vehicles increased the antimicrobial effects of CH on *E. faecalis* and other bacteria by analyzing zones of growth inhibition [41]. Notably, CH pastes with oily vehicles have been reported in the literature as medications with restricted application and in clinical situations that require a very slow ion dissociation such as permanent root filling for perforation defects after internal resorption [29,41]. Given our results, Metapex could be clinically indicated as an intracanal medication for up to 15 days with great antimicrobial performance and reduction in the extracellular biofilm matrix. The chemical mechanism by which this oil can potentiate the antimicrobial effect of CH pastes needs to be further investigated.

This is the first study to investigate the amount of extracellular biofilm matrix after applying CH pastes. This type of analysis is important because the extracellular matrix of the biofilm plays an important protective function, even when microorganisms enter a non-culturable state where they continue to express antigen components and virulence factors [13,14]. In addition, after chemomechanical procedures, the remaining biofilm can regrow depending on the environmental conditions [42]. Our results showed that UltraCal XS and Metapex have better performance in extracellular matrix reduction in direct contact and intratubular decontamination assays. CHP and Metapaste had intermediate values. However, these findings should be evaluated with caution, as to disorganizing the biofilm extracellular matrix, a mechanical procedure must be performed [13]. Antimicrobial agents cannot penetrate biofilms without the aid of physical-mechanical action, especially when the biofilms are densely organized [15,43]. Chemomechanical preparation is the main mechanism responsible for the elimination of biofilms during complete endodontic treatment [15]. Therefore, the use of intracanal medication aims to eliminate residual microorganisms from the chemomechanical preparation, partially dislodged from their biofilms in hidden ramifications or those found in the depth of the tubules, not necessarily involving large amounts of extracellular matrix.

## 5. Conclusions

None of the CH pastes maintained the pH necessary for their repair properties for up to 30 days. All CH pastes presented similar volumetric loss in 15 days, considering that this would be an adequate intermediate period between endodontic treatment sessions since the investigated CH pastes began to lose their alkalizing properties. Metapex, composed of CH with iodoform and an oily vehicle (silicone oil), generated lower values of bacterial viability in direct contact and intratubular decontamination assays. UltraCal XS and Metapex have similar performances in extracellular matrix biofilm reduction.

## Figures and Tables

**Table 1:** Median (minimum–maximum) pH values at different times after contact with calcium hydroxide pastes.

Groups	7 Days	15 Days	30 Days
Distilled water	7.03	7.03	7.03
CHP	10.5 ^Aa^ (9.80–10.8)	7.6 ^Ba^ (7.3–7.8)	7.5 ^Ba^ (7.5–7.6)
UltraCal XS	10.3 ^Aa^ (9.6–10.8)	7.5 ^Ba^ (7.4–8.2)	7.7 ^Ba^ (7.4–8.0)
Metapaste	10.6 ^Aa^ (10.2–10.7)	8.9 ^Bb^ (8.0–9.4)	8.3 ^Ba^ (7.5–8.9)
Metapex	9.40 ^Ab^ (8.90–10.5)	7.5 ^Ba^ (7.3–9.0)	6.3 ^Bb^ (5.7–6.6)

CHP, calcium hydroxide with propylene glycol. Kruskal–Wallis and Dunn’s tests (*p* < 0.05). Different capital letters represent significant differences between the evaluated periods. Different lowercase letters indicate significant differences between the four groups in a column.

**Table 2:** Mean and standard deviation (SD) values of the initial volume (mm^3^) and after 15 days of immersion of the pastes in 10 mL of distilled water, and volumetric alteration percentage (%) comparing the initial and final volumes.

Groups	Initial Volume	Final Volume	Volumetric Alteration
CHP	2.48 ^Aa^ (1.02)	2.17 ^Ab^ (1.12)	17.66 ^A^ (10.54)
UltraCal XS	2.26 ^Aa^ (0.84)	1.71 ^Ab^ (0.64)	19.46 ^A^ (12.03)
Metapaste	2.66 ^Aa^ (0.86)	1.40 ^Ab^ (0.52)	37.11 ^A^ (24.48)
Metapex	2.91 ^Aa^ (0.84)	1.69 ^Ab^ (0.56)	37.07 ^A^ (18.53)

CHP, calcium hydroxide with propylene glycol. One-way ANOVA and Tukey’s test were used (*p* < 0.05). Different capital letters represent significant differences between the groups (differences between lines). Different lowercase letters represent significant differences between the initial and final moments of volume evaluation (differences between columns).

**Table 3:** Median (minimum–maximum) percentage (%) of bacterial viability and extracellular matrix (μm^3^) after contact with calcium hydroxide pastes in dentin blocks.

Groups	Bacterial Viability	Extracellular Matrix
Control	77.9 ^a^ (48.5–96.4)	720,259 ^a^ (611,556–1,367,897)
CHP	24.4 ^ab^ (5.24–61.2)	339,421 ^ab^ (120,756–1,167,868)
UltraCal XS	7.74 ^b^ (1.29–26.0)	264,119 ^b^ (8116–782,927)
Metapaste	4.33 ^bc^ (0.41–28.9)	580,443 ^a^ (88,202–1,682,094)
Metapex	1.72 ^c^ (0.03–10.3)	145,552 ^b^ (478–1,529,176)

CHP, calcium hydroxide with propylene glycol. Kruskal–Wallis and Dunn’s tests (*p* < 0.05). Different letters represent significant differences between groups (differences between groups/lines).

## Data Availability

Data sharing is not applicable for this article.
